# Correction: Emerging insights of bile acids in cardiovascular physiology and disease: from molecular mechanisms to therapeutic potential

**DOI:** 10.3389/fphar.2026.1976192

**Published:** 2026-09-30

**Authors:** Zhou Cheng, Lianmei Xue, Miaoyu Song, Renzhen Wang, Chenyue Yuan, Hanyun Zhang, Xiaozhen Hu, Yongning Sun

**Affiliations:** Shanghai Municipal Hospital of Traditional Chinese Medicine, Shanghai University of Traditional Chinese Medicine, Shanghai, China

**Keywords:** bile acids, cardiovascular disease, FXR receptor, mechanism, TGR5 receptor, therapeutic targets

An incorrect number was provided for Shanghai Three Year Action Plan for Further Accelerating the Inheritance, Innovation and Development of Traditional Chinese Medicine (2025–2027). The correct number is ZY(2025-2027)-1-2-4.

The original version of this article has been updated.

